# Low circulating levels of the mitochondrial-peptide hormone SHLP2: novel biomarker for prostate cancer risk

**DOI:** 10.18632/oncotarget.20134

**Published:** 2017-08-10

**Authors:** Jialin Xiao, Lauren Howard, Junxiang Wan, Emily Wiggins, Adriana Vidal, Pinchas Cohen, Stephen J. Freedland

**Affiliations:** ^1^ Leonard Davis School of Gerontology, University of Southern California, Los Angeles, California, USA; ^2^ Department of Biostatistics and Bioinformatics, Duke University School of Medicine, Durham, North Carolina, USA; ^3^ Division of Urology, Veterans Affairs Medical Center, Durham, North Carolina, USA; ^4^ Department of Surgery, Center for Integrated Research on Cancer and Lifestyle, Samuel Oschin Comprehensive Cancer Institute, Cedars-Sinai Medical Center, Los Angeles, California, USA

**Keywords:** health disparity, mitochondria, humanin-like-peptide, aging, retrograde signaling

## Abstract

**Context:**

Mitochondrial DNA mutations and dysfunction are associated with prostate cancer (PCa). Small humanin-like peptide-2 (SHLP2) is a novel mitochondrial-encoded peptide and an important mitochondrial retrograde signaling molecule.

**Objective:**

To determine whether serum SHLP2 concentration is associated with PCa risk and whether associations are race-specific.

Design, Setting and Participants: Patients undergoing prostate biopsy were recruited from the Durham Veterans Affairs hospital. Serum was collected prior to biopsy and SHLP2 measured by ELISA. We selected 200 men for analysis (100 negative biopsies and 100 PCa cases; 100 black and 100 white).

**Results:**

Mean SHLP2 levels were significantly higher in white controls versus black controls and SHLP2 was significantly higher in white controls versus white PCa cases. In contrast, there was no significant difference in SHLP2 levels between black controls and black cases. SHLP2 levels > 350-pg/ml ruled out PCa with ≥ 95% accuracy in both races.

**Conclusions:**

Lower SHLP2 was linked with increased PCa risk in white men, but no significant association was observed in black men. While SHLP2 > 350-pg/ml ruled out PCa in both races with high accuracy, SHLP2 was unrelated to PCa grade. These data suggest the circulating mitochondrial-derived peptide hormone, SHLP2 plays a key role in the development and racial disparity of prostate cancer.

## INTRODUCTION

Prostate cancer (PCa) is the most common cancer in men in the United States and the second leading cause of death from cancer in men [1]. Therefore, identification of disease-specific biomarkers is critical to addressing current challenges of whom to biopsy and how to choose interventional therapies. In spite of the clear involvement of androgens and other sex steroids in the pathogenesis of prostate cancer, the levels of these hormones are not useful in screening or diagnosis [2]. Prostate-specific antigen (PSA) is an important tool for screening patients for PCa, but PSA has a number of limitations. The lack of specificity of PSA for PCa has resulted in unnecessary biopsies and an over-diagnosis of indolent PCa. In fact, only about 25% of men who undergo a prostate biopsy due to an elevated PSA level have PCa [3]. Secondly, despite a “normal” PSA, men can still harbor PCa [4]. Taken together, the development of novel biomarkers that can improve the accuracy of screening is becoming increasingly important [5].

The vertebrate mitochondrion encodes 13 important oxidative phosphorylation (OXPHOS) proteins and serves as the cellular center of energy production. In many cancer cells, “aerobic glycolysis” occurs instead of maximal ATP generation by OXPHOS, suggesting mitochondrial dysfunction. One potential source of new biomarkers may be derived from the mitochondria, given that many of the key aspects of malignancy involve changes in mitochondrial energy metabolism and resistance to mitochondrial apoptosis [6, 7]. Furthermore, specific mutation and deletion patterns in the mitochondrial DNA (mtDNA) have been associated with various types of cancer including PCa [8, 9]. However, mtDNA might not be a convenient biomarker for PCa as very little neoplastic DNA is shed from the prostate epithelium to the urine or blood, thus making the mutated mtDNA dilute and difficult to detect [10]. Moreover, mitochondrial cancer mutations must lead to altered signaling in the nucleus to have biological impact, a process called retrograde signaling [11]. Hence, the ideal PCa biomarker should be a mitochondrial retrograde signaling molecule that is directly involved in neoplastic transformation. Our group identified several peptides encoded by small open-reading-frames (sORFs) in the mtDNA [12]. These include humanin, the first mitochondrial-derived-peptide (MDP) discovered, which is encoded in the 16S rRNA region of the mtDNA, is secreted into the circulation, and acts as a cytoprotective factor via its cognate receptor, as well MOTS-c, encoded in the 12S rRNA region, which is a potent metabolo-protective factor [13–15]. We recently reported the expression and biological significance of six additional small peptides encoded within the mitochondrial 16S rRNA region, referred to as “small humanin-like peptides (SHLPs)” [16]. Among these, SHLP2 has potent retrograde signaling effects on reducing reactive oxygen species (ROS) production and improving mitochondrial metabolism *in vitro* as well as protective actions *in vivo* [16]. In addition to their retrograde signaling function, these MDPs may act as hormones, for all the currently discovered MDPs are transported by the circulatory system and actively involved in metabolism. For example, humanin regulates glucose homeostasis via hypothalamic STAT3 activation and is a new player in the growth hormone (GH)/insulin-like growth factor (IGF) axis [17–19]. MOTS-c appears to regulate glucose metabolism and muscle insulin action [15]. We believe SHLP2 also represents a novel mitochondrial hormone as it belongs to the MDP family and can influence mitochondrial respiration. Moreover, we developed a SHLP2 ELISA assay and showed that its levels decline with age [16]. Taken into account the importance of mitochondria in PCa progression and the role of SHLP2 as a mito-enhancing hormone, we hypothesized that SHLP2 may be associated with PCa risk and possibly serve as a novel biomarker for this disease.

## RESULTS

**Figure 1:**
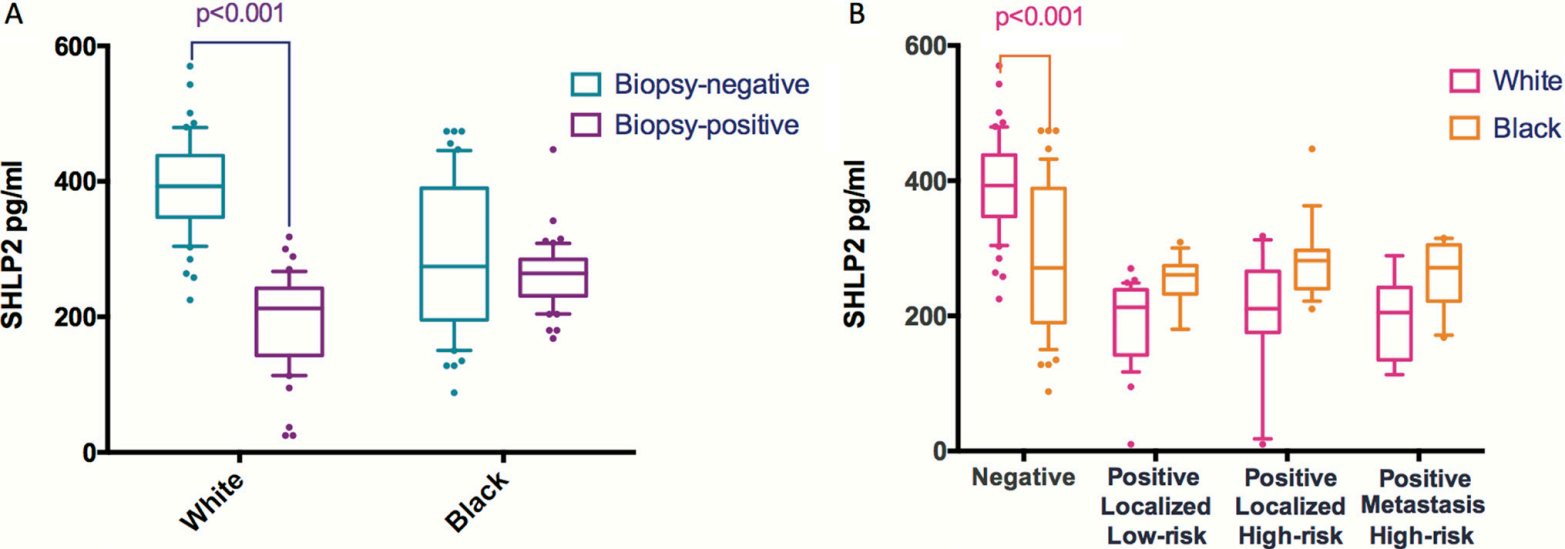
The distribution of SHLP2 levels stratified by race or outcome (**A**) When stratified by race, mean SHLP2 was significantly higher in white controls vs. white cases (393 vs. 196 pg/ml, *p* < 0.001), while the difference in SHLP2 between black controls vs. black cases was smaller and not statistically significant (289 vs. 261 pg/ml, *p* = 0.093). (**B**) When stratified by outcome, average value of SHLP2 was significantly higher in white control than black control (393 vs. 290, *p* < 0.001). However, SHLP2 levels were not different between white and black in all cancer groups.

Baseline characteristics and serum SHLP2 levels are shown in Table 1. There were no significant differences between cases and controls for age, BMI, and DRE. Of the cases, there were 63 low-grade (Gleason score ≤ 6) and 37 high-grade (Gleason score 7–10). In addition, we analyzed the associations between SHLP2 and other variables (Table 2). Men with SHLP2 ≤ 350-pg/ml were more likely to be black (*p* = 0.010), had more recent biopsies (*p* < 0.001), and higher PSA (*p* = 0.004), compared to men with SHLP2 >350-pg/ml. The serum levels of SHLP2 were higher in controls than cases (341 vs. 230-pg/ml, *p* < 0.001). When stratified by race (Figure 1A), SHLP2 was higher in white controls vs. white cases (393 vs. 196-pg/ml, *p* < 0.001), while the difference between black controls vs. black cases was smaller and not significantly different (289 vs. 261-pg/ml, *p* = 0.093). SHLP2 was similar between white and black cases (296 vs. 276-pg/ml, *p* = 0.17); however, SHLP2 was higher in white than black controls (393 vs. 290-pg/ml, *p* < 0.001) (Figure 1B). There was no association between SHLP2 and PCa grade (Figure 1B).

**Table 1:** Baseline characteristics in cases and controls

	Cases (*N* = 100)	Controls (*N* = 100)	Total (*N* = 200)	*p* value
**Age**				0.125^1^
Median	63	61.5	62	
Q1, Q3	60, 66	59, 65	59, 66	
**Race**				0.888^2^
White	49 (49%)	50 (50%)	99 (50%)	
Black	51 (51%)	50 (50%)	101 (50%)	
**Year of consent**				< 0.001^1^
Median	2010	2008	2008	
Q1, Q3	2009, 2011	2008, 2008	2008, 2010	
**BMI (kg/m2)**				0.150^1^
Median	28.3	29.8	28.9	
Q1, Q3	25.4, 31.3	25.9, 32.9	25.6, 32.1	
**PSA (ng/ml)**				< 0.001^1^
Median	7.1	5.5	6.0	
Q1, Q3	5.0, 13.4	4.4, 7.5	4.7, 9.1	
**Grade group**				
Missing	0	100	100	
1	63 (63%)	0 (0%)	63 (63%)	
1	14 (14%)	0 (0%)	14 (14%)	
2–3	23 (23%)	0 (0%)	23 (23%)	
**DRE**				0.404^2^
Normal	75 (75%)	81 (81%)	156 (78%)	
Suspicious	24 (24%)	19 (19%)	43 (22%)	
Unknown	1 (1%)	0 (0%)	1 (1%)	
**Prostate Volume (cc)**				0.009^1^
Median	36.5	45.5	40.0	
Q1, Q3	27.0, 56.8	34.0, 69.5	28.5, 63.1	
**Family history of PC**				< 0.001^2^
No	54 (54%)	78 (78%)	132 (66%)	
Yes	20 (20%)	17 (17%)	37 (19%)	
Unknown	26 (26%)	5 (5%)	31 (16%)	
**Diabetes**				0.893^2^
No	51 (51%)	54 (54%)	105 (53%)	
Yes	17 (17%)	15 (15%)	32 (16%)	
Unknown	32 (32%)	31 (31%)	63 (32%)	
**SHLP2**				< 0.001^1^
Median	241	361.5	268.5	
Q1, Q3	205, 270	261, 421.5	218, 363	
**SHLP2**				< 0.001^2^
< = 350	99 (99%)	43 (43%)	142 (71%)	
> 350	1 (1%)	57 (57%)	58 (29%)	

^1^Wilcoxon ^2^Chi-Square.

**Table 2:** Association between SHLP2 and other variables (SHLP2 cut-off at 350-pg/ml)

	< = 350 (*N* = 142)	> 350 (*N* = 58)	*p* value
**Age**			0.335^1^
Median	63	61	
Q1, Q3	59, 66	59, 65	
**Race**			0.010^2^
White	62 (44%)	37 (64%)	
Black	80 (56%)	21 (36%)	
**Year of consent**			< 0.001^1^
Median	2009	2008	
Q1, Q3	2008, 2011	2007, 2008	
**BMI (kg/m2)**			0.709^1^
Median	28.4	29.6	
Q1, Q3	26.0, 32.0	25.2, 32.8	
**PSA (ng/ml)**			0.004^1^
Median	6.6	5.0	
Q1, Q3	4.9, 9.5	4.4, 7.4	
**Grade group**			
No cancer	43	57	
1	63 (64%)	0 (0%)	
2–3	13 (13%)	1 (100%)	
4–5	23 (23%)	0 (0%)	
**DRE**			0.802^2^
Normal	111 (78%)	45 (78%)	
Suspicious	30 (21%)	13 (22%)	
Unknown	1 (1%)	0 (0%)	
**Prostate Volume (cc)**			0.180^1^
Median	38.0	44.5	
Q1, Q3	27.7, 62.4	31.4, 70.0	
**Family history of PC**			0.077^2^
No	87 (61%)	45 (78%)	
Yes	29 (20%)	8 (14%)	
Unknown	26 (18%)	5 (9%)	
**Diabetes**			0.884^2^
No	73 (52%)	32 (55%)	
Yes	23 (16%)	9 (16%)	
Unknown	46 (32%)	17 (29%)	
**Status**			< 0.001^2^
Controls	43 (30%)	57 (98%)	
Cases	99 (70%)	1 (2%)	

^1^Wilcoxon ^2^Chi-Square.

**Figure 2:**
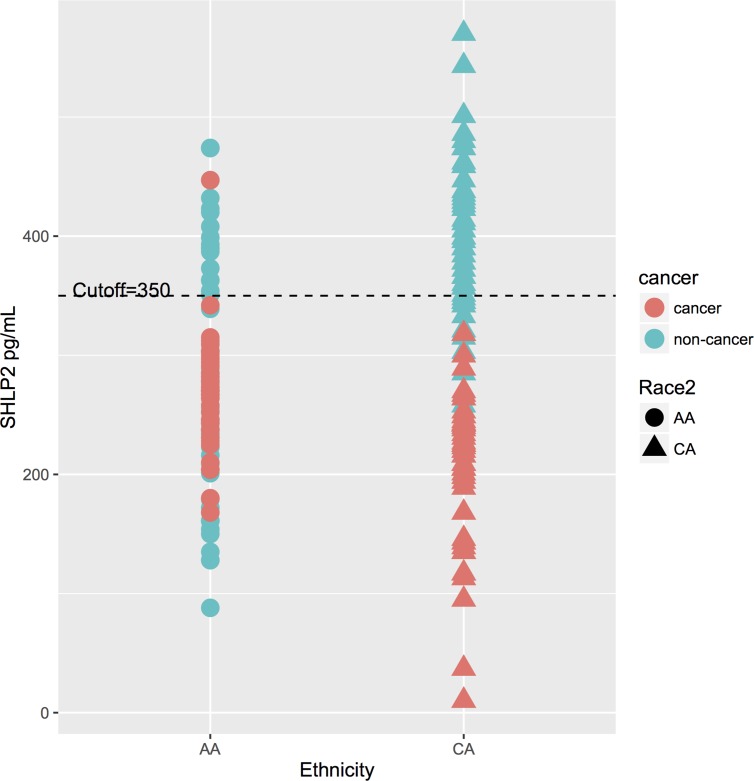
The distribution of SHLP2 levels and a cut-off at 350-pg/ml A cut-off of 350-pg/ml SHLP2 differentiate between controls and PCa cases in both black and white men. Among men with SHLP2 > 350-pg/ml, 0/37 white (100% NPV) and only 1/20 black men had PCa (95% NPV).

The p-interaction between race and SHLP2 for predicting PCa was 0.006. Thus, analyses were stratified by race. On multivariable analysis, lower SHLP2 was linked with increased risk of overall, low-, and high-grade PCa in white men (all *p* < 0.038), but none of these associations even approached significance in black men (all *p* > 0.37) (Table 3). After adjusting for family history of PCa and prostate volume, results were similar in black men but stronger in magnitude among white men, albeit the associations had larger confidence intervals and were not statistically significant with the exception of high-grade PCa (Supplementary Table 1). A SHLP2 cut-off of 350-pg/ml separated PCa from controls in both black and white men (Figure 2). Among men with SHLP2 > 350-pg/ml, 0/37 white (100% NPV) and only 1/20 black men had PCa (95% NPV), which was a Gleason 7. Using a SHLP2 cut-off > 350-pg/ml to not biopsy would have avoided 57/100 negative biopsies while missing one Gleason 7.

**Table 3:** Association between SHLP2 and overall risk of cancer and risk of cancer grade, stratified by race

	OR	White	*P*-value	OR	Black	*P*-value
		95% CI			95% CI	
Univariable						
No PC	Ref.			Ref.		
Overall PC	0.95	0.93–0.97	< 0.0001	0.996	0.99–1.00	0.095
No PC	Ref.			Ref.		
Low-grade PC	0.95	0.93–0.97	< 0.001	0.99	0.90–1.00	0.044
High-grade PC	0.95	0.93–0.97	< 0.001	1.00	0.99–1.00	0.598
Multivariable*						
No PC	Ref.			Ref.		
Overall PC	0.92	0.84–0.995	0.037	0.998	0.99–1.01	0.706
No PC	Ref.			Ref.		
Low-grade PC	0.91	0.83–0.99	0.038	1.00	0.98–1.01	0.377
High-grade PC	0.91	0.83–0.99	0.031	1.00	0.99–1.02	0.671

*Adjusted for age, BMI, PSA, year of biopsy, and digital rectal exam.

**Figure 3:**
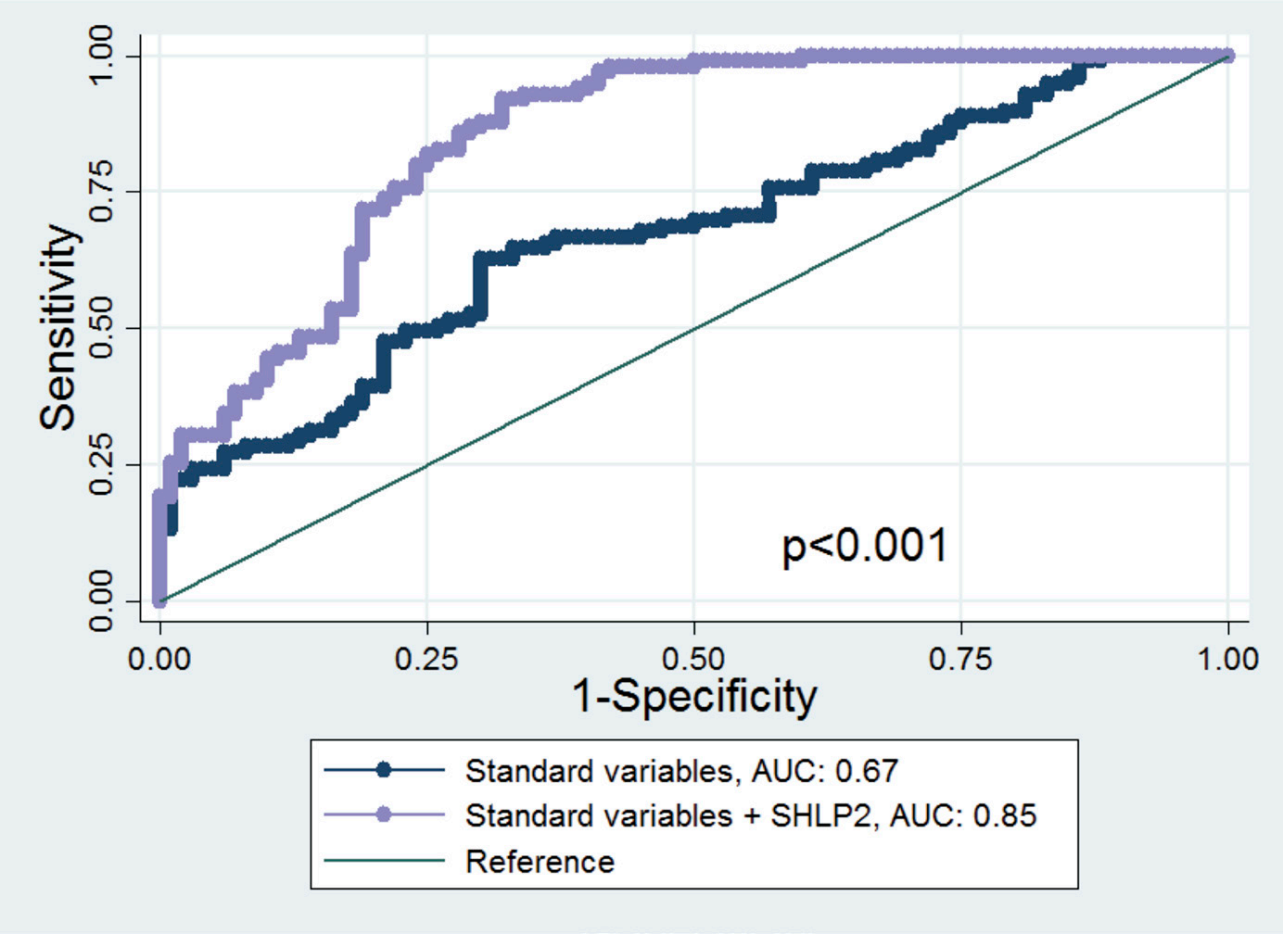
ROC curve and AUC statistics before and after adding SHLP2 in the model The true positive rate (sensitivity) is plotted in function of the false positive rate (1−specificity) for the model excluding or including SHLP2 levels. The AUC is a measure of how well a quantitative test can distinguish between subjects with and without prostate cancer. The AUC of the model including only age, DRE, race and PSA to predict PCa risk was 0.67. This improved to 0.85 when SHLP2 was added to the model (*p* < 0.001).

The AUC of the model including only age, DRE, race and PSA (standard clinical variables) to predict PCa risk was 0.67. This improved to 0.85 when SHLP2 was added to the model (*p* < 0.001). Moreover, there was a substantial improvement in AUC for white men (0.72 to 0.99, *p* < 0.001) but only a slight increase in AUC for black men (0.70 to 0.72, *p* = 0.47) after SHLP2 was added to the model including age, DRE, and PSA (Figure 3).

## DISCUSSION

Cancer cell metabolism has attracted increasing attention as a promising area of cancer study. One of the first pieces of evidence suggesting a link between mitochondrial metabolism and cancer is the excessive production of lactate and glycolytic metabolism of tumor tissues [20]. Later studies were carried out to determine whether this aerobic glycolysis was due to mitochondrial OXPHOS dysfunction, which generally yielded negative results [21]. In addition to mtDNA mutations/deletions detected in cancer cells, mutations in nuclear-encoded mitochondrial enzymes are also associated with tumorigenesis [22]. These findings suggest neoplastic transformation taking place in mitochondria do not only involve defects in mitochondrial energy production but also altered mitochondrial bioenergetics and metabolomics. As for PCa, the aggressive phenotypes of cancer cells are associated with metabolic transformation to aerobic glycolysis, citrate oxidizing, and loss of ability to accumulate zinc [23, 24]. Therefore, identifying the key signaling events leading to reprogrammed metabolism in cancer cells and the tumor environment might be more useful for developing cancer therapeutics and reliable biomarkers. Our group has been focusing on the mitochondrial retrograde signaling and how it can impact metabolism. Previously reported mitochondrial retrograde signals include small molecules such as Ca^2+^, reactive oxygen species and NADH. We further investigated mtDNA for the possibility of translating small peptides from sORFs, and identified several hormone-like peptides with retrograde signaling functions. These MDPs are secreted in circulation and play important roles in the cell.

SHLP2 activates ERK and STAT3 signaling pathways via an unknown receptor. It is a regulator for mitochondrial respiration and ROS production. Moreover, administration of SHLP2 improved insulin sensitivity in rodent models [16]. In this case-control study, we demonstrated that serum levels of the mitochondrial protein SHLP2 > 350 pg/ml are strongly predictive of a negative biopsy in both black and white men undergoing prostate biopsy. While we have no direct explanation for the relationship between SHLP2 and cancer development, and our results require confirmation in other studies, if validated the observed association between SHLP2 and PCa risk suggests a possible role for SHLP2 in the development of PCa. SHLP2 is both mitochondrially encoded and regulates mitochondrial function; therefore, its levels may be an indicator of mitochondrial integrity and health, preventing carcinogenesis. As such, low SHLP2 levels may represent a more generalized mitochondrial dysfunction that could represent a pre-malignant state.

There are several alternative hypothetical explanations for the relationship between low SHLP2 and PCa that we can speculate about. First, during cancer development, the prostate microenvironment co-evolves with the tumor and facilitates the acquisition of its malignant phenotype. It is possible that the low levels of SHLP2 contribute to a favorable milieu for tumor growth by shifting cells towards aerobic glycolysis [25–27]. Moreover, the shift in mitochondrial metabolism and energetics may contribute to the cancer-initiating characteristic of prostate cancer stem cells [28]. Previous study also demonstrated that the mtDNA-depleted prostate cancer cells exhibit cancer stem cell features [29]. Second, chronic inflammation is closely associated with PCa etiology. SHLP2 promotes mitochondrial function and may support healthy immune surveillance as well as suppress pro-inflammatory signaling. Since SHLP2 has been shown to reduce mitochondrial ROS [16], lower SHLP2 levels may lead to elevated oxidative stress, which could potentially be a major source of mitochondrial genome instability leading to mitochondrial dysfunction. As a result, mitochondrial dysfunction could activate the redox-sensitive NF-kB and trigger the release of inflammatory cytokines [30]. Alternatively, SHLP2 may lower PCa risk through its effects on enhancing insulin sensitivity. Insulin resistance is known to be associated with a higher risk of PCa, and may exacerbate cancer through upregulation of inflammation, oxidative stress and unbound testosterone [31]. Therefore, the insulin sensitizing action of SHLP2 may contribute to its protective effects against PCa. The continuous intra-cerebro-ventricular (ICV) infusion of SHLP2 improved hepatic glucose metabolism and peripheral glucose uptake, which indicates that this centrally acting peptide also triggers a change in peripheral insulin responsiveness [16]. Although the signaling events linking SHLP2 and glucose metabolism are unknown, it is possible that this humanin-like molecule activates STAT3 and AKT pathways [18]. Furthermore, SHLP2 maintains mitochondrial integrity and may prevent insulin resistance by reducing mitochondrial ROS generation. PCa risk has been reported to be related to circulating IGF-I levels [32], which are inversely related to the levels of the mitochondrial peptide humanin [33]. As SHLP2 and humanin share similarities, it is also possible that higher SHLP2 levels are correlated with lower IGF-I levels, thus predicting lower PCa risk. Our observation of differential regulation of SHLP2 levels in black and white men coupled with the known differential risk for PCa incidence and mortality rates between races is intriguing.

Endocrine involvement in PCa has been well recognized [34], with sex steroids, growth factors, and cytokines playing a role. The involvement of mitochondria in this disease is also recognized [35], and the current study implicates mitochondrial-derived peptides. An intriguing finding is that the black controls have lower SHLP2 levels than white controls. It is possible that the lifestyle differences between races, such as exercise and diet composition, can affect mitochondrial functions and influence metabolism. The lower SHLP2 levels of black controls might be due to impaired mitochondrial activities. As for genetic factors, the different mitochondrial haplogroups not only identify major branches points on the phylogenetic tree, but also demonstrate differences in OXPHOS activities, mtDNA copy number and mitochondrial-encoded gene expression as evidenced by a number of cybrid studies [36]. Moreover, mtDNA of black men may harbor SNPs that directly suppress SHLP2 expression. In fact, health disparity among black and white men with prostate cancer has always been an issue. The racial difference of SHLP2 levels in control men may provide partial explanation to PCa ethnic disparity. Furthermore, evidence for the contribution of mtDNA to prostate cancer has been shown to involve mutations and variations in the region of the mtDNA RNR2/16SrRNA which is near the site of the SHLP2 ORF [8]. Since the causes of PCa involve genetic, hormonal and environmental elements, additional studies are underway to further discern the relationship of SHLP2 levels with other PCa risk factors, including obesity and diabetes, that have been linked to mitochondrial peptides [37]. Further investigations are needed to understand the biological role of SHLP2 in PCa development and progression.

Our study is not without limitations. The number of men included was small and thus validation is needed. While SHLP2 was not significantly related to PCA risk as a continuous variable for black men, using a cut-off of > 350-pg/ml it had a 95% NPV. If validated, this suggests examining SHLP2 using cut-off values may have more clinical utility than as a continuous variable, though we lacked sufficient power to explore multiple cut-points. As such, future studies are needed to both validate our findings and explore potentially alternative cut-points. Future studies are needed to explore how SHLP2 correlates with other hormonal levels thought to be important for PCa such as testosterone. Our outcome was PCa on biopsy. As some men with a negative biopsy may still harbor PCa, there will be some misclassification. Though SHLP2 did not correlate with grade, further evaluation of SHLP for other end-points including progression, which was not available in our dataset, is needed. Future studies are needed to explore the role of SHLP2 in races other than white and black men.

In conclusion, our data suggest the mitochondrial peptide SHLP2 may be a novel PCa biomarker. Future studies are needed to confirm these findings and understand the mechanistic link between low SHLP2 and PCa as well as the link between SHLP2 and race and whether it is involved in explaining racial disparities in PCa.

## MATERIALS AND METHODS

### Subjects

We used samples from an ongoing case-control study of men undergoing prostate biopsies at the Durham Veterans Affairs Medical Center. The study was approved by the Institutional Review Board and written informed consent was obtained from all subjects before enrollment. Subjects were recruited between January 2007 and September 2015 from the urology clinic. Eligible subjects were men with no prior history of PCa who were undergoing a prostate needle biopsy because of abnormal PSA and/or suspicious digital rectal exam (DRE). We selected 200 men with available serum samples for our study equally divided by biopsy outcome and race (100 negative biopsies; 100 PCa cases; 101 black and 99 white). Diabetes status and race were self-reported.

### Biochemical analysis of serum

Serum was collected from all patients prior to the biopsy. Most patients (*n* = 148; 74%) were fasting at the time of blood draw, but as SHLP2 levels were similar in those fasting vs. not fasting (*p* = 0.078), fasting was not considered in the models. Endogenous serum SHLP2 levels were measured using our developed SHLP2 ELISA using total immunoglobin (Ig)G and ligand purified antibodies with a detection limit of 50 pg/ml. Custom rabbit anti-SHLP2 antibody was ordered from Harlan (Indianapolis, IN). The intra- and inter-assay coefficient variations (CV) of the SHLP2 ELISA were less than 10%. Prior to the SHLP2 ELISA, from each sample, 100 μL of serum was extracted using an acid solution (90% acetonitrile and 10% 1 N HCl). The supernatant was dried using a SpeedVac™ (Thermo Fisher, Waltham, MA). The dried samples were reconstituted with assay buffer (50 mM PBS containing 0.5% Tween 20). 96-well microtiter plates were coated with SHLP2 capture antibody at a concentration of 0.5 μg/well in 200 μL of 50 mM sodium bicarbonate buffer (pH 9.5). The plates were incubated for 3–4 h at room temperature on a shaker, washed with wash buffer, and then washed twice with Superblock™ buffer (Pierce Chemicals, Rockford, IL). Standards, controls, or extracted samples were added to the appropriate wells with pre-tittered detection antibody and incubated overnight. After washing, streptavidin-HRP (horse radish peroxidase) was added and further incubated for 30 min at room temperature. After four washes with wash buffer, 200 μL/well of OPD (o-phenylenediamine dihydro-chloride) substrate (1 mg/ml in hydrogen peroxide) was added and incubated for 10–20 minutes. Reactions were terminated with 50 μL/well 2 N H_2_SO_4_, and absorbance values were measured on a plate spectrophotometer (Molecular Designs, Sunnyvale, CA) at 490 nm.

### Statistical analyses

Wilcoxon or chi-squared tests were used for univariable comparisons of subject characteristics between cases and controls, and also between those with SHLP2 ≥ 350 pg/ml vs. < 350 pg/ml. The interaction between SHLP2 and race in predicting PCa was tested by including a cross product term in a logistic regression model. Logistic regression models to test the association between SHLP2 and PCa risk were then stratified by race. Multinomial logistic regression was used to test the link between SHLP2 and low-grade PCa (Gleason < 7) vs. no PCa and high-grade PCa (Gleason 7–10) vs. no PCa. Models were adjusted for age, BMI, PSA (logarithmically transformed), DRE and year of biopsy. In a secondary analysis, we also adjusted for prostate volume and family history of PCa.

A SHLP2 cut-off of 350 pg/ml (∼upper tertile) was used to assess the negative predictive value (NPV) of PCa by race. ROC curves were plotted as false-positive rate (1–specificity) vs sensitivity for adjusted SHLP2 values. The diagnostic performance of SHLP2 to predict prostate biopsy results (our primary outcome, PCa vs no cancer) in each race group was assessed using the area under the ROC curve (AUC). Statistical analyses were performed using SAS^®^ 9.3 (SAS Institute Inc.; Cary, NC) and Stata^®^ 13.1 (StataCorp.; College Station, TX), with *p* < 0.05 defined as statistical significance.

## SUPPLEMENTARY MATERIALS TABLE



## Abbreviations

PCa: prostate cancer
SHLP2: small humanin-like peptide 2
MtDNA: mitochondrial DNA
sORF: small open-reading-frame
MOTS-c: mitochondrial open reading frame of the 12S rRNA-c
ROS: reactive oxygen species
DRE: digital rectal exam
NPV: negative predictive value
AUC: area under curve
