# Supplementary material for: Low circulating levels of the mitochondrial-peptide hormone SHLP2: novel biomarker for prostate cancer risk
Source: Oncotarget. 2017 Aug 10;8(55):94900–9. doi: 10.18632/oncotarget.20134 (PMC5706922; doi:10.18632/oncotarget.20134)
Supplement: Supplementary file 1 [file oncotarget-08-94900-s001.pdf]

Low circulating levels of the mitochondrial-peptide hormone SHLP2: novel biomarker for prostate cancer risk

SUPPLEMENTARY MATERIALS

Supplementary Table 1: Association between SHLP2 and overall risk of cancer and risk of cancer grade, stratified by race

	White			Black		
	OR	95% CI	*P*-value	OR	95% CI	*P*-value
**Multivariable***						
**No PC**	Ref.			Ref.		
**Overall PC**	0.82	0.65–1.03	0.090	1.00	0.99–1.01	0.592
**No PC**	Ref.			Ref.		
**Low-grade PC**	0.80	0.64–1.00	0.053	1.00	0.98–1.01	0.458
**High-grade PC**	0.80	0.64–0.996	0.046	1.00	0.99–1.02	0.606

*adjusted for age, BMI, PSA, DRE, year of consent, prostate volume, and family history of prostate cancer.
